# Scurvy: A New Old Cause of Skeletal Pain in Young Children

**DOI:** 10.3389/fped.2020.00008

**Published:** 2020-01-31

**Authors:** Christel Chalouhi, Nayla Nicolas, Nancy Vegas, Soraya Matczak, Houmam El Jurdi, Nathalie Boddaert, Véronique Abadie

**Affiliations:** ^1^General Pediatrics Department, Necker Hospital, Paris, France; ^2^Pediatric Imaging Unit, Necker Hospital, Paris, France; ^3^Université de Paris – Faculté de Médecine Paris Descartes, Paris, France

**Keywords:** vitamin C, ascorbic acid, scurvy, selective diet, skeletal pain

## Abstract

We report 3 cases of scurvy in children that occurred during a short period (2018) in a general pediatrics unit of a tertiary hospital for children in Paris. All children were around 3 years of age and were admitted for skeletal pain and altered general state, which mimicked infectious or malignant diseases. Their selective diet was not the prominent issue. The diagnosis of scurvy was delayed, after too many unnecessary examinations and medications. Bone imaging findings (X-ray and MRI) were *a posteriori* considered typical, but lesions were not easily identified as scurvy lesions because scurvy is not well-known by pediatricians and radiologists who should be mindful of this historical diagnosis.

## Introduction

Modern-day physicians tend to consider scurvy a disease of the past, first described in ancient times, as illustrated by the writings of Hippocrates (460 BC): “… *the breath smells bad, the gums separate from the teeth, blood runs from the nostrils, black-colored ulcerations frequently appear on the legs, some heal others do not, and the skin is thin…”*. Similar descriptions are found in the writings of Jean de Joinville, a counselor to Louis IX of France, during the Seventh Crusade in Egypt in 1248–1254. More familiarly, the great navigators who underwent long expeditions (Vasco de Gama, 1497) suffered the first scurvy outbreaks. During the Renaissance, Jacques Henri Bernardin de St Pierre, aboard an East India Company ship in 1768, wrote: “*the first sign of scurvy is general lethargy: the patient craves rest; feels disgruntled; is sickened by everything; suffers during the day; only finds solace at night; then red dots appear on the legs and chest and bleeding ulcers appear on the gums. Frequently there are no outward signs at all, but the slightest cut remains incurable while we are at sea and progresses most rapidly. I sustained a slight cut to the end of my finger. Within 3 weeks the wound had ravaged the whole finger and had already spread to my hand despite all attempts to heal it. Several days after I had reached my destination it healed of its own accord.”* In 1747, aboard the Salisbury, the surgeon James Lind undertook what is considered one of the first clinical trials: 12 patients with scurvy were administered different remedies, yet only those fed lemon or orange juice recovered (1–3). Francis Glisson first described scurvy in children in 1650, and found that it often occurred in addition to rickets. At the end of the nineteenth century, infantile scurvy resurged with the advent of heated milk and proprietary foods as popular infant foods, until Hess recommended that infants receiving heated milk should also receive fresh fruits. This recommendation led to the eradication of infantile scurvy in developed countries (4).

The present literature contains accounts of vitamin C deficiency in children in impoverished countries, notably a review published in Thailand in 2003 describing 28 children (mean age 29 months) with a diagnosis of scurvy, who had been fed a diet of ultra-high-temperature pasteurized milk combined with cooked meat and starchy food but no fresh fruit or vegetables (5). The same year, a non-governmental organization reported a hemorrhagic fever epidemic in Afghanistan that in fact proved to be a scurvy outbreak (6). Despite this knowledge, a scurvy outbreak recently occurred in a refugee camp in Kenya in 2018 (7). In developed countries, scurvy is rare and mainly described in children with autism or neurological problems.

Here we present three cases that occurred during the same year, 2018, in children presenting to the general pediatrics unit of a tertiary hospital in a socially advantaged district of Paris, in children with no special needs.

## Case 1

**Figure 1:**
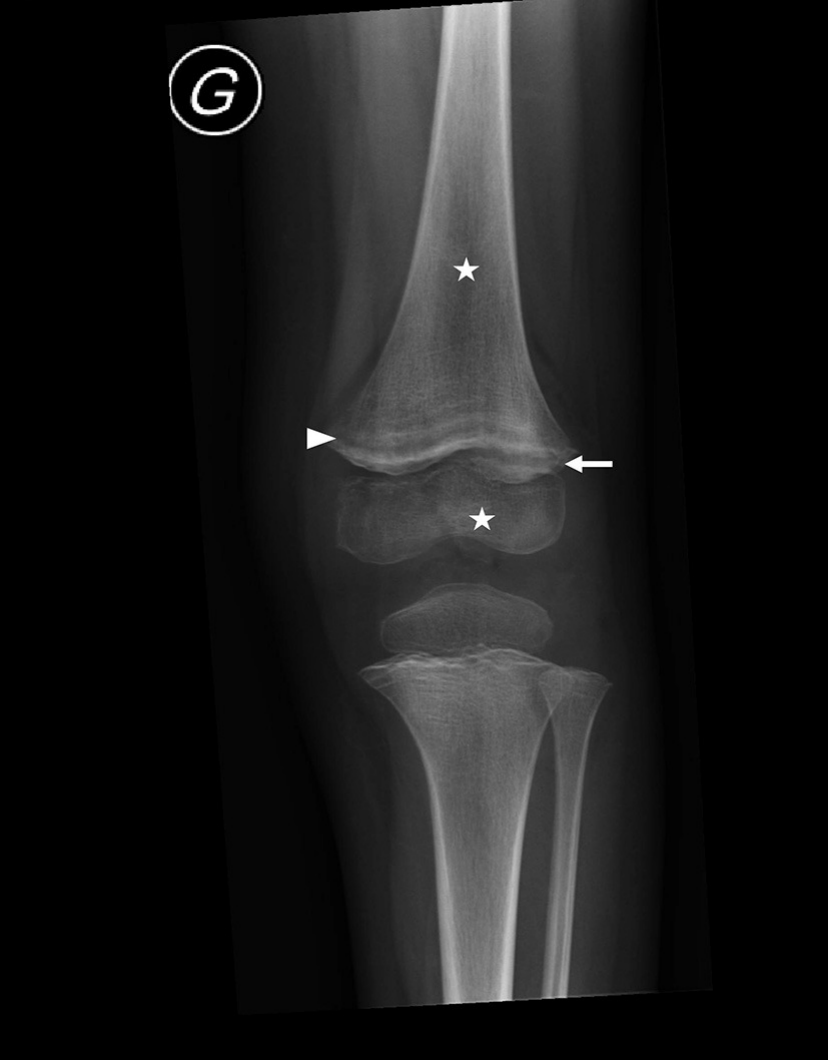
Left knee radiograph for case I, a 3-year-old boy, demonstrating multiple clear metaphyseal bands in the distal femur (white arrowhead), also defined as “Trummerfeld zone,” and marked white line corresponding to thickened zone of calcification, also named “white line of Frankel.” Irregular and enlarged metaphyseal margin (white arrow), as well as diffuse osteopenia (stars) are also noted.

A 3-year-old boy was admitted to the hospital for stunted growth, regression of walking, eating disorders and behavior troubles. He was exclusively breastfed for the first 12 months of his life, during which the transition to bottle-feeding with formula milk was unsuccessful. Therefore, breastfeeding was continued until 22 months of age, combined with an undiversified diet consisting of stewed fruits and dairy products. His initial psychomotor development was normal, but from 2 years of age, he became introverted and excessively irritable. His diet became more and more selective. During his third year of life, his growth rate had slowed. He sustained recurrent ear-nose-throat infections. One month before admission, his general condition had deteriorated; he was dejected and reluctant to walk, retreating into withdrawal behavior and playing somewhat repetitive games. After the boy was admitted to our department, we observed a malnourished and anxious child, refusing to walk. He ate only bread and goat's cheese. Clinical examination revealed conjunctival hyperemia involving bleeding from the lateral angle of the eye and erythematous gums that we attributed to an intercurrent viral infection. The initial suspected diagnosis was malignant disease and autism spectrum disorder. The septic workup was negative. Lower-limb radiographs revealed diffuse osteopenia associated with alternating dense and lucent metaphyseal bands and widening of the distal extremity of the femur (Figure 1).

In parallel, we searched for consequences of his selective diet but did not *a priori* think that all his symptoms could match scurvy. A nutritional assessment revealed low ferritin level (8 μg/L, *N* = 15–80) and null plasma vitamin C content. On this basis, classical scurvy involving gingivitis, conjunctival hemorrhage and bone damage was diagnosed but only 10 days after admission. Vitamin C treatment was rapidly effective. The boy regained motor function, and his general condition greatly improved. The diagnosis of autism spectrum disorder was ruled out.

## Case 2

**Figure 2:**
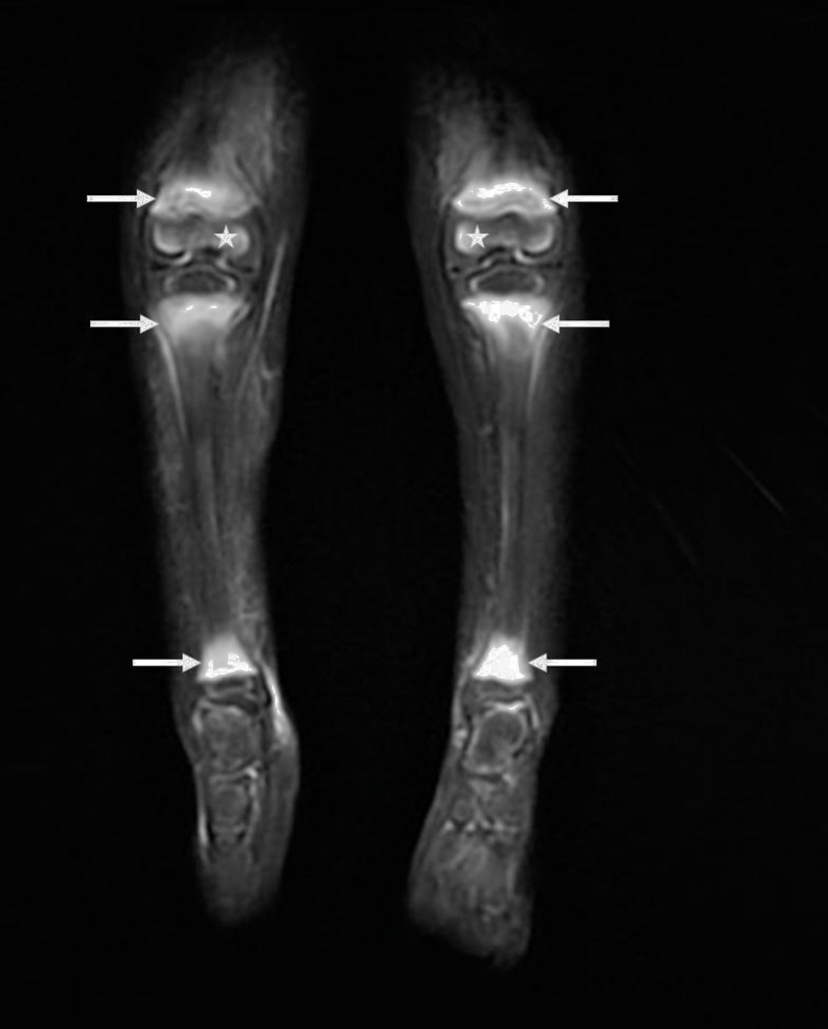
Bilateral lower leg MRI for case 2, a 3.5-year-old girl: coronal fat-suppressed T2-weighted image revealing bilateral bone-marrow edematous changes of the metaphyseal area (white arrows) of the ankles and knees. Edematous changes were also noted in the distal epiphysis of the femur bilaterally (stars). Whole-body MRI shows multiple similar anomalies in the wrists and shoulders.

A 3.5-year-old girl was admitted to hospital for pain on walking and persistent crying. She was the first child of healthy parents. Her appetite was low, yet growth was normal and her parents reported no restrictive eating behavior. Development was normal until 2 years of age, but for 6 months, she had been repeatedly complaining of leg pain and had intermittent limping that was attributed to transient synovitis or “growing pains.” Two months before admission, the girl began to complain of left knee pain, and her appetite was suppressed, and she had become increasingly fatigued and irritable. Her diet had become increasingly selective, consisting of starchy food, dairy products and bread. We observed an introverted, anxious child who was prone to tears and easily distressed, especially when approached or touched. Objective assessment was normal in all other respects. Osteomyelitis was initially suspected despite no biological inflammatory reaction. She received an 8-day course of antibiotics. In light of persistent signs and a negative septic workup, leukemia was suspected. However, bone-marrow aspiration was normal. Initial radiograph findings showed mild vertebral osteopenia at T11, and MRI revealed symmetric bone-marrow signal changes in the metaphysis of long bones, more prominent in the lower extremities. These findings are compatible with malignancy, rheumatologic process such as chronic recurrent multifocal osteomyelitis or nutritional deficiency (Figure 2).

We suspected a chronic recurrent multifocal osteomyelitis and started anti-inflammatory treatment. During a staff meeting when the case was presented, the physician who had taken care of the first case described the above evoked scurvy. Plasma vitamin C content was severely deficient in the girl (0.8 μmol/L, normally 40–100 μmol/L) and confirmed scurvy but only 10 days after admission. Ferritin level was in the low range of standard (28 μg/l). Vitamin C treatment resulted in rapid reduction of all symptoms.

## Case 3

**Figure 3:**
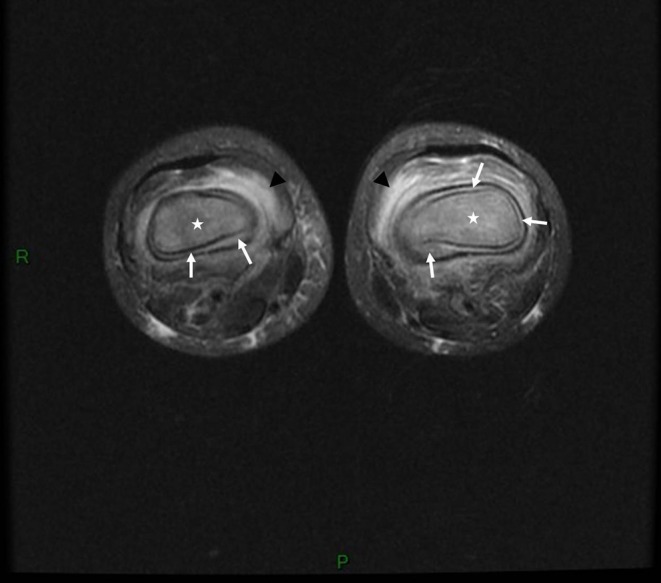
Bilateral knee MRI for case 3, a 3-year-old girl: axial fat-suppressed T2-weighted image showing marked bilateral edematous bone-marrow changes within (stars) and around the bone (black arrowheads) associated with bilateral circumferential sub periosteal collections (white arrows) related to bilateral sub-periosteal hematomas.

A 3-year-old girl was admitted to hospital for functional impairment in both lower limbs, with fever and deteriorated general physical condition. She was a twin, born at 34 weeks' gestation. Because of the parents' inadequate care, she and her sister were placed in foster care at birth, where they remained until age 7 months, when were returned to their family. Their development was satisfactory for the first year of life, but then the girl progressively developed an eating disorder, becoming selective. She would eat only a particular brand of creamy yogurt and a brand of mixed baby food. Although the quality of her diet was suboptimal, her quantity of intake seemed adequate, and she was growing well. At 8 days before admission, she was seen for lower-limb pain, fever and an inability to walk. Tests searching for infection yielded negative findings and the girl was discharged with a diagnosis of probable viral disease. The parents returned 3 days later because of persistent symptoms. The girl was admitted for evaluation. We observed an introverted, very unsociable and apprehensive girl, who lay flat in her stroller. She had dry skin and inflammatory hair follicles. Laboratory tests showed no inflammatory reaction. Bone radiographic findings were unremarkable; however, MRI of the lower limbs revealed multifocal symmetric bone-marrow signal anomalies within the metaphysis associated with circumferential sub-periosteal collections around both femoral metaphysis. Edematous signal changes were also noted in the deep and superficial tissues surrounding the elevated periosteum. These bone lesions immediately evoked scurvy (Figure 3). Plasma vitamin C content was severely deficient (0.4 μmol/L). Ferritin level was normal (66 μg/l). As noted in the previous cases, within days, vitamin C treatment had a positive effect on pain, behavior, and motor function.

## Comments

These 3 cases confirm that scurvy is a re-emerging disease that pediatricians need to consider, especially in the presence of unexplained bone pain, even in children with typical development. These three children, especially the child described in case 2, would have had fewer investigations if the diagnosis of scurvy had been considered earlier. Doctors must ask about dietary habits, especially in children around 3 years of age who are no longer receiving infant formula but who may not yet have a diversified diet.

Vitamin C or ascorbic acid is an essential vitamin since the occurrence of a genetic mutation inhibiting the conversion of glucose to ascorbic acid in primates 40 million years ago made humans dependent on an exogenous supply of vitamin C. This enzyme is still present in most mammals (8, 9). Vitamin C is very actively absorbed by the ileum and rapidly eliminated by renal excretion. With excessive intake, it is absorbed passively and also excreted renally with no risk of overdose. However, insufficient intake owing to low reserves results in onset of biological deficiency within 3 months and clinical signs within 6 months (10). Ascorbic acid plays a critical role in the formation of type II collagen, and deficiency accounts for bone and vessel wall lesions. Ascorbic acid has antioxidant and anti-infection properties and enhances non-heme iron absorption and heavy metal detoxification (11).

Classical signs of scurvy include gingival lesions together with inflammation, hypertrophy and loosened teeth resulting from bleeding. Oral lesions accentuate anorexia, leading to food selectivity. Second-stage disorders are of a cutaneous nature, involving dry skin, folliculitis, vascular purpura and hematomas that can be painful, thus accentuating anorexia. Third-stage disorders are of a musculoskeletal nature, involving osteoporosis, bone growth abnormality, and subperiosteal or intraosseous hemorrhagic lesions that mimic inflammatory disease. Radiographic findings suggestive of scurvy are located in the metaphysis of all long bones, more prominent in the lower limb and include a clear metaphyseal band, also named “Trummerfeld zone;” marked white line corresponding to thickened zone of calcification also named “white line of Frankel;” irregular metaphyseal margin or metaphyseal fractures known as “Pelkan spur;” and diffuse osteopenia. MRI findings are concordant with radiographic findings: multifocal, symmetric bone-marrow changes in the metaphysis of long bones. When these features are associated with multifocal circumferential periosteum elevation, scurvy must be ruled out (12). Fourth-stage disorders affect the general physical condition, with asthenia and dejection that probably also perpetuate eating disorders. Of note, the symptoms leading to hospitalization in the 3 cases were skeletal symptoms.

The US recommended daily intake of vitamin C is 15 mg/day for children 1 to 3 years old, 25 mg/day for those 4 to 8 years old, approximately 50 mg/day for those 8 to 18 years old and 100 mg for adults. The richest food sources of vitamin C are fruit and vegetables, especially blackcurrants, parsley, peppers, lemons, lychees, strawberries, raspberries, gooseberries, papaya and kiwis (13).

In France, as in other developed countries, 3 sets of circumstances can lead to the re-emergence of scurvy. The first represents adults living in highly precarious conditions. The second is children whose selective diet is totally devoid of fresh fruit and vegetables. Such eating behaviors may occur in children with autism spectrum disorders or severe neurodevelopmental disabilities but also in children with aversion restrictive food intake disorders or only very selective eating behavior. Their diets must be totally devoid of fruits and vegetables, not only poor intake, as is commonplace. The third scenario is children fed a diet of vegetable beverage such as almond milk or other types of beverage popular with vegan parents. For these children, assessment of their nutritional status must be performed. Of note, in our cases, iron deficiency was present only in one out of three.

About 80 pediatric cases have been published in the past 20 years, essentially occurring in children with autism spectrum and neurodevelopmental disorders (14–23) but not necessarily (24–26). Vitamin C deficit is more frequent than classical scurvy, as recently shown by a retrospective review of the files of children with low ascorbic level (27). Further accounts are available on the Internet and in the general press.

## Conclusion

Risk of scurvy continues to exist, even in developed countries, in individuals lacking vitamin C intake for more than 3 to 6 months. Therefore, caution is warranted for all children whose eating habits for whatever reason, are selective. For these selective-eating toddlers, in addition to psycho-educational rehabilitation, young children formula can be continued after 3 years of age, and vitamin C must be given as orange juice or vitamin supplements. Scurvy symptoms should be known to pediatricians. Similarly, pediatric radiologists should be mindful of scurvy, given that presentations frequently mimic infectious or malignant bone abnormalities.

## Data Availability Statement

The raw data supporting the conclusions of this article will be made available by the authors, without undue reservation, to any qualified researcher.

## Ethics Statement

Written informed consent was obtained from the minor(s)' legal guardian/next of kin for the publication of any potentially identifiable images or data included in this article.

## Author Contributions

CC, NV, HE, and SM are the pediatricians who cared for the children. NN and NB are the radiologists who helped us for the diagnosis of scurvy and selected the images. VA was the head of the general pediatric unit where the children were admitted. VA, CC, and HE wrote the manuscript. All the authors corrected the manuscript and validated the last version.

### Conflict of Interest

The authors declare that the research was conducted in the absence of any commercial or financial relationships that could be construed as a potential conflict of interest.

## References

[1] Still GF A clinical lecture on infantile scurvy. Br Med J. (1906) 28:186–90. 10.1136/bmj.2.2378.186 PMC2381834 20762798

[2] Still GF. Infantile scurvy: its history. Arch Dis Child. (1935) 10:211–8. 10.1136/adc.10.58.211 21031997 PMC1975442

[3] Jacques Henry Bernardin de Saint Pierre Voyage à l'île de France un officier du roi à l'Ile Maurice (1768–1770). Merlin: Paris (1773).

[4] Rajakumar K. Infantile scurvy: a historical perspective. Pediatrics. (2001) 108:E76. 10.1542/peds.108.4.e76 11581484

[5] Ratanachu EKS Sukswai P Jeerathanyasakun Y Wongtapradit L Scurvy in pediatric patients: a review of 28 cases. J Med Assoc Thai. (2003) 86 (Suppl. 3):S734–40. 14700174

[6] Cheung E Mutahar R Assefa F Ververs MT Nasiri SM Borrel A. An epidemic of scurvy in Afghanistan: assessment and response. Food Nutr Bull. (2003) 24:247–55. 10.1177/156482650302400303 14564929

[7] Ververs M Muriithi JW Burton A Burton JW Lawi AO Scurvy outbreak among south sudanese adolescents and young men - kakuma refugee camp, Kenya, 2017-2018. Morb Mortal Wkly Rep. (2019) 25:72–5. 10.15585/mmwr.mm6803a4 PMC6348760 30677009

[8] Montel-Hagen A Kinet S Manel N Mongellaz C Prohaska R Battini JL Red cell GLUT1 compensates for the lack of vitamin C synthesis in mammals. Med Sci. (2008) 2:434–6. 10.1051/medsci/2008244434 18405646

[9] Yang H Conserved or lost: molecular evolution of the key gene GULO in vertebrate vitamin C biosynthesis. Biochem Genet. (2013) 51:413–25. 10.1007/s10528-013-9574-0 23404229

[10] Jacob R Vitamin C. In: Shils M Olson J Shike M Ross AC, editors. Vitamins. Modern Nutrition in Health and Disease. Philadelphia, PA: Lippincott (2000). p. 467.

[11] Figueroa-Méndez R Rivas-Arancibia S. Vitamin C in health and disease: its role in the metabolism of cells and redox state in the brain. Front Physiol. (2015) 6:397. 10.3389/fphys.2015.00397 26779027 PMC4688356

[12] Niwa T Aida N Tanaka Y Tanaka M Shiomi M Machida J. Scurvy in a child with autism: magnetic resonance imaging and pathological findings. J Pediatr Hematol Oncol. (2012) 34:484–7. 10.1097/MPH.0b013e318236c519 22258350

[13] Olson JA Hodges RE. Recommended dietary intakes (RDI) of vitamin C in humans. Am J Clin Nutr. (1987) 45:693–703. 10.1093/ajcn/45.4.693 3565296

[14] Agarwal A Shaharyar A Kumar A Bhat MS Mishra M. Scurvy in pediatric age group - A disease often forgotten? J Clin Orthop Trauma. (2015) 6:101–7. 10.1016/j.jcot.2014.12.003 25983516 PMC4411344

[15] Swed-Tobia R Haj A Militianu D Eshach O Ravid S Weiss R. Highly selective eating in autism spectrum disorder leading to scurvy: a series of three patients. Pediatr Neurol. (2019) 94:61–3. 10.1016/j.pediatrneurol.2018.12.011 30795887

[16] Ceglie G Macchiarulo G Marchili MR Marchesi A Rotondi Aufiero L Di Camillo V. Scurvy: still a threat in the well-fed first world? Arch Dis Child. (2019) 104:381–3. 10.1136/archdischild-2018-315496 30087152

[17] Rafee Y Burrell K Cederna-Meko C. Lessons in early identification and treatment from a case of disabling vitamin C deficiency in a child with autism spectrum disorder. Int J Psychiatry Med. (2018) 54:64–73. 10.1177/0091217418791443 30079810

[18] Kinlin LM Blanchard AC Silver S Morris SK. Scurvy as a mimicker of osteomyelitis in a child with autism spectrum disorder. Int J Infect Dis. (2018) 69:99–102. 10.1016/j.ijid.2018.02.002 29425711

[19] Burhop J Gibson J de Boer J Heydarian C. Do you C what I C: emergency department evaluation and diagnosis of pediatric scurvy in an autistic child with a restricted diet. Pediatr Emerg Care. (2020) 36:e1–3. 10.1097/PEC.0000000000001412 29369263

[20] Planerova A Philip S Elad S. Gingival bleeding in a patient with autism spectrum disorder: a key finding leading to a diagnosis of scurvy. Quintessence Int. (2017) 48:407–11. 10.3290/j.qi.a38060 28396889

[21] Seya M Handa A Hasegawa D Matsui T Nozaki T. Scurvy: from a selective diet in children with developmental delay. J Pediatr. (2016) 177:331. 10.1016/j.jpeds.2016.06.018 27426835

[22] Ma NS Thompson C Weston S. Brief report: scurvy as a manifestation of food selectivity in children with autism. J Autism Dev Disord. (2016) 46:1464–70. 10.1007/s10803-015-2660-x 26590972

[23] Khan N Furlong-Dillard JM Buchman RF. Scurvy in an autistic child: early disease on MRI and bone scintigraphy can mimic an infiltrative process. BJR Case Rep. (2015) 1:20150148. 10.1259/bjrcr.20150148 30363625 PMC6180829

[24] Cain M Harris M Kim K Homme JH Ascorbic acid deficiency (Scurvy) in a toddler with restricted dietary intake presenting with “Leg Weakness” and a Rash. Infant Child Adolescent Nutrition. (2014) 6:201–4. 10.1177/1941406414532685

[25] Brambilla A Pizza C Lasagni D Lachina L Resti M Trapani S. Pediatric scurvy: when contemporary eating habits bring back the past. Front Pediatr. (2018) 6:126. 10.3389/fped.2018.00126 29780794 PMC5946015

[26] Hahn T Adams W Williams K Is vitamin C enough? a case report of scurvy in a five-year-old girl and review of the literature. BMC Pediatr. (2019) 19:2–6. 10.1186/s12887-019-1437-3 30849951 PMC6408840

[27] Golriz F Donnelly LF Devaraj S Krishnamurthy R. Modern American scurvy - experience with vitamin C deficiency at a large children's hospital. Pediatr Radiol. (2017) 47:214–20. 10.1007/s00247-016-3726-4 27778040

